# Modelling potential habitat for snow leopards (*Panthera uncia*) in Ladakh, India

**DOI:** 10.1371/journal.pone.0211509

**Published:** 2019-01-29

**Authors:** Sophie M. Watts, Thomas M. McCarthy, Tsewang Namgail

**Affiliations:** 1 Snow Leopard Conservancy India Trust, Leh, Ladakh, India; 2 Panthera, New York, United States of America; Sichuan University, CHINA

## Abstract

The snow leopard *Panthera uncia* is an elusive species inhabiting some of the most remote and inaccessible tracts of Central and South Asia. It is difficult to determine its distribution and density pattern, which are crucial for developing conservation strategies. Several techniques for species detection combining camera traps with remote sensing and geographic information systems have been developed to model the habitat of such cryptic and low-density species in challenging terrains. Utilising presence-only data from camera traps and direct observations, alongside six environmental variables (elevation, aspect, ruggedness, distance to water, land cover, and prey habitat suitability), we assessed snow leopard habitat suitability across Ladakh in northern India. This is the first study to model snow leopard distribution both in India and utilising direct observation data. Results suggested that elevation and ruggedness are the two most influential environmental variables for snow leopard habitat suitability, with highly suitable habitat having an elevation range of 2,800 m to 4,600 m and ruggedness of 450 m to 1,800 m. Our habitat suitability map estimated approximately 12% of Ladakh's geographical area (c. 90,000 km^2^) as highly suitable and 18% as medium suitability. We found that 62.5% of recorded livestock depredation along with over half of all livestock corrals (54%) and homestays (58%) occurred within highly suitable snow leopard habitat. Our habitat suitability model can be used to assist in allocation of conservation resources by targeting construction of livestock corrals to areas of high habitat suitability and promoting ecotourism programs in villages in highly suitable snow leopard habitat.

## Introduction

Conservation of threatened species requires accurate knowledge of their distributions so that conservationists and managers can delineate and optimise protected areas on a priority basis [1]. Determining distributions is crucial for long-term survival of threatened species in the face of increasing anthropogenic pressure on natural areas [2]. This is particularly important when considering the conservation of apex predators as they are often considered keystone, umbrella, or flagship species, and their protection can benefit the entire ecosystem [3]. Snow leopards are one such example of a flagship species [4], and are listed as vulnerable by the International Union for Conservation of Nature (IUCN [5]) due to three major threats of anthropogenic origin: natural prey depletion due to competition with domestic livestock, retaliatory killing following livestock depredation, and poaching to fuel the illegal trade in fur and bones [5].

Due to their elusiveness and low density in the landscape, snow leopards’ distribution and abundance have been preferentially assessed through non-invasive techniques such as interviews, sign surveys (scats, rock scents, scrapes and pugmarks), and snow tracking [6–11]; methods that can be deployed over a large area at relatively low field cost and effort [12]. Camera-trapping has been used to study snow leopard's habitat affinities, and density patterns [13]. While sightings of other species by wildlife managers and more recently citizen scientists have contributed to species distribution and abundance estimates [14,15], hitherto no study used direct observation of snow leopards for modelling its distribution largely due to the difficulty in sighting them in the field. By analysing available spatial data on species presence in combination with environmental variables, spatially explicit models can be used to identify species habitat preferences and predict distributions [16–20]. Species distribution models (SDM) and habitat suitability (HS; a measure of the ability of a habitat to sustain a species [1,21]) index models in conservation biology and wildlife management [18,22–25] play a critical role in conservation decision-making [20].

SDMs for carnivores can be difficult to model appropriately as their elusiveness results in challenges obtaining suitable data [26]. Fortunately, the existence of presence-background (often referred to as presence-only) modelling methods facilitates SDMs or HS modelling of understudied and elusive species [27–29]. One such method is maximum entropy (MaxEnt) modelling, by finding the most uniform probability distribution, that can be used where data are sparse or irregular to estimate species’ niche distribution [30,31]. MaxEnt models have been considered the best for predictive performance of SDMs [31], while also facilitating a measure of habitat suitability. Several felid population distributions have been predicted using MaxEnt (e.g. [2,32]) including two subspecies of leopard [33,34].

Of the big cat species, snow leopards *Panthera uncia* are the least studied [13], in part due to their rugged, remote habitat [6,35–37] at altitudes typically between 3,000 m and 4,500 m [38]. Using various modelling approaches, snow leopard habitat has already been assessed across its entire geographic range that spans 12 central and south-Asian countries covering 2.8 million km^2^ [39]; however, regional assessment and mitigation efforts are required to allocate conservation resources [40]. Despite this, regional habitat assessment has so far only been carried out in Nepal [41] and two reserves in China; Sanjiangyuan National Nature Reserve (SNNR [42,43]) and Qomolangma National Nature Reserve (QNNR [44]).

The Indian Himalayas is estimated to support between 500 and 700 snow leopards yet the snow leopard distribution in India has only been estimated using the Digital Chart of the World at a scale of 1:1,000,000 [45]. Sixty percent of India’s snow leopards are thought to be in Ladakh [22], where conservation investments include strengthening livestock corrals [26], livestock insurance schemes, and providing a supplementary income associated with an increase in ecotourism such as community-managed homestays [46] and handicrafts sales [47,48]. This highlights to local people the intrinsic and economic value of snow leopards, and reduces retaliatory killing [49,50]. In order to identify and best target these conservation investments in Ladakh, this study aimed to produce a visualisation of snow leopard habitat suitability by utilising camera trap and direct observation data in MaxEnt software.

## Materials and methods

### Ethics statement

All the techniques used to study the animals were non-invasive. Permission to conduct camera trap surveys was obtained from the Department of Wildlife Protection (CFWLL/E.P./2011/91-93; CFWLL/E.P./2012/956-58). Direct observation data of snow leopards and ungulate prey were obtained on public land, and thus no approval was required. Depredation data were analysed retrospectively using data obtained by SLC-IT during depredation surveys.

### Study area

**Fig 1:**
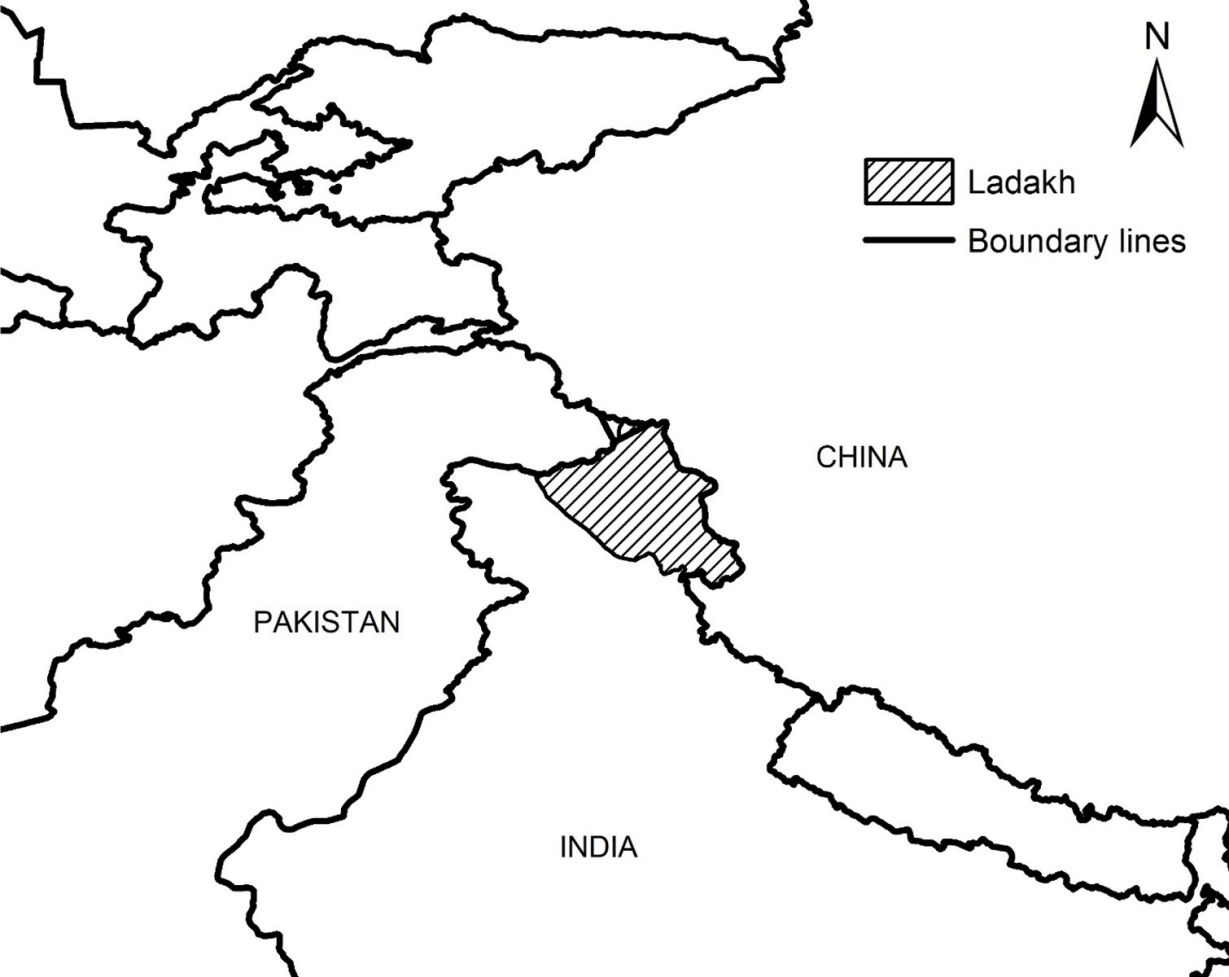
Location of Ladakh in northern India with Pakistan to the northwest and China to the northeast.

Ladakh (75°50’E—80°E and 32°30’N—37°N; Fig 1), located in the northern Indian state of Jammu & Kashmir, extends over approximately 90,000 km^2^ with the presence of the Trans-Himalayan mountain range resulting in elevations of 2,600 m to 7,200 m. Ladakh is defined as a high-altitude cold desert with an average annual precipitation of less than 200 mm and mid-winter snow depths of only 10 cm in central Ladakh [35]. Vegetation is a combination of steppe vegetation and shrubland [51] that sustain populations of four wild sheep and goats; Asiatic ibex *Capra ibex sibirica*, blue sheep *Pseudois nayaur*, Tibetan argali *Ovis ammon hodgsoni* and Ladakh urial *Ovis vignei vignei* of which the first two are most preferred by snow leopards.

### Presence data

Data on snow leopard presence were compiled from camera trap surveys between 2011 and 2016, and direct observations between 2010 and 2017. Over this time period, camera traps were set up within grid cells of 4 x 4 km in four major valleys. The target cells containing physical features preferred by snow leopards were selected randomly. Snow leopard images were captured spanning the entire calendar and were georeferenced. Direct observations of snow leopards however occurred in two major valleys during the winter months, which coincides with mating season and increased snow leopard activity [40]. During this season, snow leopards also descend to lower areas following the natural prey species and livestock [52]. Snow leopards were located by scanning the mountain slopes in early morning and late evening using binoculars and spotting scopes. All direct observations were recorded with an approximate position based on observer location, and bearing and distance to the cat. Snow leopards were sighted in different parts of Ladakh, and thus the direct observations are considered spatially representative. Auto-correlation presence locations were removed resulting in a total of 167 unique snow leopard presence locations (63 camera traps and 104 direct observations). Camera traps and direct observations were aggregated for better coverage.

Presence data of the two main prey species (Asiatic ibex and blue sheep) were also recorded during the described camera trapping surveys and from opportunistic direct observations between 2011 and 2018. We recorded 97 unique ungulate presence locations (19 camera traps and 78 direct observations).

We used SDMtoolbox [53] in ArcGIS 10.3.1 to spatially filter presence locations within 1 km of each other. The remaining 84 ungulate presence points (11 from camera traps and 73 from direct observations) and 83 snow leopard presence points (46 from camera traps and 37 direct observations) were used in model construction (S1 and S2 Files).

### Environmental variables

To reduce the risk of over-fitting, considering our small sample size, and to construct the most parsimonious model, we included environmental variables based on a priori knowledge of snow leopard habitat preference [52,54]. We calculated elevation, slope, aspect and ruggedness using a digital elevation model (DEM) in ArcGIS 10.3.1. Terrain ruggedness (TRI) was determined by calculating the elevation difference between adjacent cells of the DEM [55]. We obtained land cover data from the University of Louvain and European Space Agency’s GlobCover 2009 (http://due.esrin.esa.int/page_globcover.php) and annual mean temperature from (www.worldclim.org [56]). Path distance to water sources (river/stream/lake) and roads were also calculated using data from Open Street Map (www.openstreetmap.org), and GPS locations of villages were obtained during routine field visits. All raster layers were clipped to the extent of Ladakh and resampled at 1 km resolution to correspond with the original resolution of WorldClim data [56]. The value of each environmental variable was extracted at presence locations and following multicollinearity analysis, correlated variables were removed (r^2^ ≥ 0.75). We ran a preliminary model for overall prey habitat suitability (Asiatic ibex and blue sheep combined) within MaxEnt version 3.4.1 [30] that was later included as an environmental variable (prey habitat suitability; S1 Fig). Six environmental variables (one biotic and five abiotic); elevation, aspect, ruggedness, distance to water, land cover, and prey habitat suitability were used in the snow leopard analysis.

### Modelling

We input all environmental variables and snow leopard locations into MaxEnt version 3.4.1. [30]. We ran the model through ten replications using the Bootstrap method, with 75% training data and 25% for model validation [31]. Default settings were utilised, and output was logistic. Jackknife and sensitivity analyses were carried out on each variable to determine its contribution and importance to the model. Area Under the receiver operator characteristic Curve (AUC) was used to assess model accuracy [57]. The output was interpreted as habitat suitability index [58,59] and the logistic probability was re-classified in ArcGIS to represent snow leopard habitat suitability (0.0–0.14 low; 0.14–0.42 moderate; 0.42–1.0 high).

### Conservation applications

To demonstrate the conservation applicability of the model, we considered two major components in human-snow leopard interaction; livestock depredation and snow leopard tourism. All interaction variables were spatially linked to the nearest village location, we then extracted the corresponding habitat suitability and other environmental variables from ArcGIS.

Snow leopard depredation events (n = 80) between 2009 and 2012 were recorded during interviews with villagers across Ladakh. Locations of predator-proof corrals (n = 130) constructed with support from the Snow Leopard Conservancy–India Trust (SLC-IT) and Panthera from 2000 to 2017 were also utilised. To consider snow leopard tourism we compiled a record of known homestays (n = 125) facilitated by SLC-IT and Panthera. Chi-squared goodness of fit tests were carried out to examine whether the frequencies of depredation events, corrals, homestays, and number of associated villages were different from expected based on the number of villages in different levels of snow leopard habitat suitability.

## Results

Snow leopard presence points ranged in elevation from 2965 m to 5831 m, with a ruggedness of 250 m to 1509 m, and maximum distance to water of 1575 m. The aspect of these points ranged from 22.7° to 342°; however only two points (2.4%) were classified as north-facing (0° - 22.5°, 337.5° - 360°). Presence points only occurred in three landcover classes; closed to open herbaceous vegetation (75%), bare areas (24%), and permanent snow and ice (1%). All snow leopard presence points were found in areas of medium (22%) to high (78%) habitat suitability for prey.

Elevation was identified as the most important environmental variable (Table 1), followed by ruggedness. Landcover and aspect were considered to contribute less to the model, and landcover and prey were the least important.

**Table 1:** Contribution and permutation importance values of each environmental variable in the snow leopard habitat suitability model.

Variable	Percent contribution	Permutation importance
Elevation	70.7	63.7
Ruggedness	12.4	17.1
Water	6.3	11.9
Prey	5.3	1.8
Aspect	2.8	4.8
Landcover	2.6	0.8

Due to the exclusion of correlated variables in our model, we were able to make inferences from the marginal response curves (S6 Fig) that indicate the relationship between each environmental variable and snow leopard habitat suitability when all environmental variables except the variable of interest retain their average sample value. Notably elevations of approximately 2,900 m to 4,500 m, areas of ruggedness over 590 m, non-north-facing slopes, and any level of prey habitat suitability could be considered highly suitable habitat for snow leopard when other environmental variables took their average sample value. For land cover, all categories could result in highly suitable habitat when other environmental variables were averaged, although the most preferred landcover class was 140 (closed to open herbaceous vegetation) that covers roughly half the area of Ladakh. Finally, 0 m to 1,050 m to the nearest water source was required for habitat to be classified as highly suitable.

When each environmental variable was considered in isolation, similar inferences could be drawn for elevation and aspect (S7 Fig). Differences were evident from the other four environmental variables such that highly suitable habitat would only occur where; landcover was 140 (closed to open herbaceous vegetation) or 200 (bare areas), distance to water was less than 450 m, ruggedness was 600 m to 1,230 m, and the area was also considered highly suitable for the main prey species.

**Fig 2:**
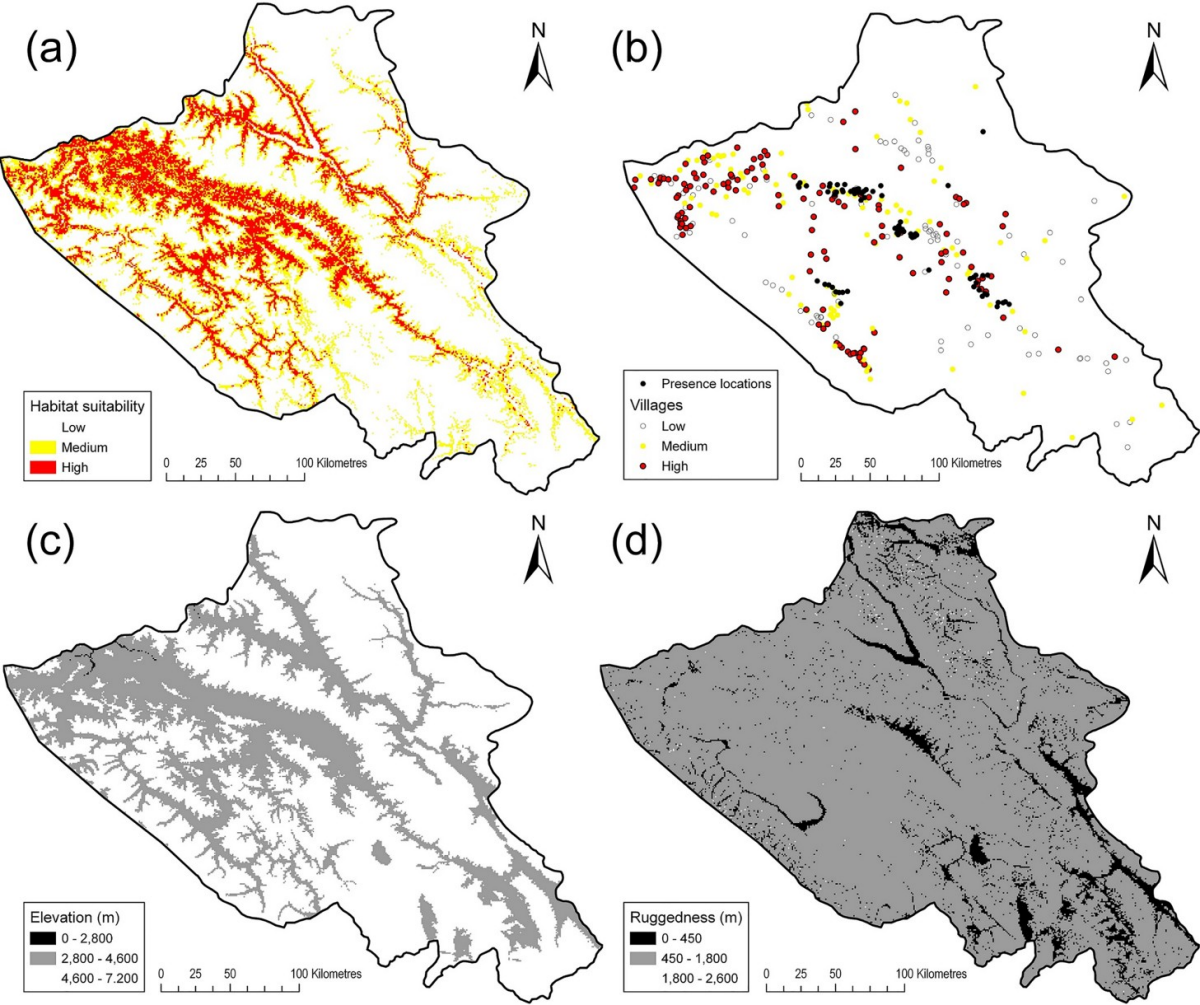
Spatial visualisation of snow leopard habitat suitability model and key environmental variables in Ladakh. (a) Distribution of presence locations (black points; n = 83) and villages across high (red points; n = 131), medium (yellow points; n = 104) and low (white points; n = 90) snow leopard habitat suitability. (b) Snow leopard habitat suitability map. (c) Elevation categorised into below optimal (black; 0 m– 2,800 m), optimal (grey; 2,800 m– 4,600 m), and above optimal (white; 4,600 m– 7,200 m). (d) Ruggedness categorised into below optimal (black; 0 m– 450 m), optimal (450 m– 1,800 m), and above optimal (white; 1,800 m– 2,600 m).

Evaluation of the MaxEnt ROC results showed a mean ± one standard deviation AUC value of 0.909 ± 0.017 indicating the model performed well with high accuracy. The reclassified mean and standard deviation from MaxEnt’s logistic output suggested approximately 62,600 km^2^ ± 6,400 km^2^ (70% ± 7%) of Ladakh is low suitability snow leopard habitat, 16,000 km^2^ ± 2,400 km^2^ (18% ± 3%) is medium suitability, and 10,800 km^2^ ± 4,000 km^2^ (12% ± 4) is high suitability snow leopard habitat (Fig 2A). From this map we can see that the area of snow leopard habitat suitability is greatest in the western half of Ladakh and reduces towards the eastern areas. Finer explanatory details can be observed in Fig 2C and 2D as the eastern side of Ladakh has larger areas of higher than optimal elevation as well as lower than optimal ruggedness.

We identified the range of values for each environmental variable in areas of high habitat suitability suggest that areas of highly suitable snow leopard habitat in Ladakh can have elevations of 2,800 m to 4,600 m, ruggedness of 450 m to 1,800 m, distance of 0 m to 1250 m to water, any aspect, all landcover classes but predominantly 140 (closed to open herbaceous vegetation; 70%) and areas of high habitat suitability for prey (90%).

Over half of the predator-proof corrals (54.6%) and 62.5% of the recorded depredation events (incidence of livestock depredation) between 2009 and 2012 occurred (Table 2) in areas of highly suitable snow leopard habitat. Homestays also exist predominantly in highly suitable snow leopard habitat, with 55.2% of the villages in this habitat category housing 58% of the homestays.

**Table 2:** Number (and percentage) of villages, villages with depredation, depredation events, villages with SLC-IT predator-proof corrals, and number of SLC-IT predator-proof corrals in high, medium, and low suitability habitats for snow leopards.

	Low (%)	Medium (%)	High (%)	Total
Village	90 (27.7)	104 (32.0)	131 (40.3)	325
Village with depredation	0 (0.0)	7 (50.0)	7 (50.0)	14
Depredation events	0 (0.0)	30 (37.5)	50 (62.5)	80
Village with corral	8 (15.7)	21 (41.2)	22 (43.1)	51
Corral	27 (20.8)	32 (24.6)	71 (54.6)	130
Village with homestay	2 (6.9)	11 (37.9)	16 (55.2)	29
Homestays	8 (6.4)	44 (35.2)	73 (58.4)	125

There was a significant difference in the observed frequency of depredation events and number of corrals (Chi-squared test: χ^2^_2_ = 32.68, P < 0.01 and Chi-squared test: χ^2^_2_ = 11.07, P < 0.01 respectively) compared to the expected frequency based on the percentage of villages found in low, medium and high suitability habitats (27.7%, 32% and 40.3% respectively); however, there was not a significant difference in the observed number of villages with corrals or that experienced depredation compared to the expected frequency based on the percentage of villages in each habitat suitability class.

There was a significant difference in the observed number of homestays and villages with homestays (Chi-squared test: χ^2^_2_ = 31.02, P = 0.01 and Chi-squared test: χ^2^_2_ = 6.43, P = 0.05 respectively) compared to the expected frequency based on the percentage of villages in each habitat suitability class.

## Discussion

In our model, elevation had the strongest influence on snow leopard habitat suitability, as was found to be the case in other regional models [12,42,44,52]. Previously, snow leopards have been shown to prefer areas of high elevation [12,52] with optimal elevation as 3,000 m to 4,500 m [38], which fits within both response curves to elevation (S6 and S7 Figs) and the range identified within highly suitable habitats in Ladakh. Elevation has been considered a limiting factor to snow leopard habitat suitability historically [52,60,61]; however, elevation itself may not directly impact habitat suitability. Elevation is negatively correlated with annual mean temperature, and thus is thought to influence habitat suitability indirectly through the altitudinal temperature gradient [41]; therefore, it could also be inferred that annual mean temperature was most important for snow leopard habitat suitability [41] as such available snow leopard habitat will likely reduce under future climate change scenario [41,61]. We elected to include elevation as its data are more widely available and can be immediately accessed in the field using a GPS, thus increasing the usability of our model; however further habitat suitability modelling under climate change scenarios may be useful.

We identified ruggedness as the second most important factor in snow leopard habitat suitability, which is consistent with regional models for SNNR [4,42] and QNNR [44]. The highly suitable snow leopard habitat in Ladakh has a ruggedness of 450 m to 1,800 m. In practice, this level of ruggedness is typical of ridgelines with a steep slope on either side or areas of rocky outcrop, which snow leopards are known to utilise [35–37] for vital shelter and potential denning sites [44]. These rugged corridors have also been associated with high densities of sign [35,36], thus facilitating communication and subsequent breeding success. In addition, presence of other carnivores such as the Tibetan wolf *Canis lupus chanko*, wild dogs *Cuon alpinus*, and feral dogs *Canis lupus familiaris* in the wider landscape, may encourage snow leopards to select more rugged areas to minimise competition [36].

While the blue sheep and Asiatic ibex occur in rugged areas [62] that snow leopards favour [12,36], they often venture on to the nearby open slopes to feed [62]. Our preliminary habitat suitability assessment for blue sheep and Asiatic ibex suggested highly suitable habitat could have a ruggedness of 20 m to 2,500 m; however, this environmental variable contributed the least and was of lowest importance in model construction (S1 Table). This mismatch of habitat requirements is particularly pronounced in simple mountain ecosystems where the predators, in this case snow leopards, are often considered as habitat specialists and are thus well adapted to marginal habitats [63]. Consequently, prey habitat suitability was considered of intermediate importance and contribution to our final model construction. The weak influence of prey on snow leopard habitat suitability has previously been explained as the result of a trade-off between physical habitat features such as elevation or ruggedness with food availability [54]; snow leopards preferentially utilise the rocks and ridgelines of highly rugged landscapes to stalk prey, while the ibex and blue sheep were observed mostly when they were feeding on open slopes [60]. Nevertheless, carnivore distribution cannot be considered independently from prey [64–66], thus when prey was the sole environmental variable considered in model construction only areas of high prey habitat suitability could be classified as highly suitable snow leopard habitats (S7 Fig). In combination, this overall reliance on prey for survival [64–66] as well as reduction in natural prey densities due to habitat loss and overgrazing by livestock [38] may result in snow leopards undergoing prey switching [67] or continue to increase the proportion of livestock into their diet.

The three remaining environmental variables of lower contribution and importance were landcover class, distance to water, and aspect. The importance of landcover is likely to be an indirect consequence of the importance of vegetation for ungulate prey (S1 Table). As landcover classes are broadly classified, pattern of vegetation along river courses was not strongly distinguished, however this in addition to the valuable provision of water and their potential as travel corridors and marking sites [36] may have all contributed to the gradual decline in habitat suitability away from water. The inclusion of this distance variable may have resulted in potential sampling bias in our model that is incurred by the ease of access associated with rivers/streams, roads, and human settlements [68]. We aimed to reduce spatial bias by spatially filtering data within 1 km from each other, as the habitat was considered heterogenous [69]. This ensured enough data for modelling and we did not observe an indication of spatial-autocorrelation in the output test omission lines [70].

The final environmental variable to consider was aspect, that became a limiting factor of habitat suitability on north-facing slopes, which may be another indirect relationship caused by differences in vegetation [71], harsh weather conditions, or in relation to prey (S2 and S3 Figs).

Regarding conservation applications of our model, we considered depredation and snow leopard tourism. We indicated that the frequency of depredation events was significantly different to those expected, with the greatest contributing difference being the lower observed depredation in areas of low suitability and higher observed depredation in highly suitable areas. As 62.5% of the recorded depredation events occurred in areas of highly suitable snow leopard habitat, we consider the predicted map (Fig 2) to be realistic. When considering villages that experienced depredation, we found an insignificant difference between observed and expected frequencies. We attribute this to the fact that snow leopards inhabiting those areas are likely to return to the same village for multiple hunting trips, for example one village experienced 15 separate depredation events.

To date, over half of the constructed corrals (54.6%) occur in highly suitable snow leopard habitat; however, some villages contain multiple corrals (22 villages in highly suitable habitats have 71 corrals). Over 20% of corrals occur in areas of low habitat suitability for snow leopards, where no depredation events have been recorded in this study; therefore, here there is scope to utilise the habitat suitability model for better targeting of conservation investment in the future. By prioritising corral construction in villages within highly suitable snow leopard habitat, the benefits will be greater.

In addition, the investment in and benefits from snow leopard tourism could also be influenced by our habitat suitability model. Already there is a significant difference between observed and expected frequency of homestays and villages that house them, with over half located in highly suitable habitat. These homestays are largely favoured by tourists in the summer due to their proximity to trekking routes as well as the mountainous environment that lends itself to snow leopards; however, winter snow leopard tourism is focused around only two valleys. Snow leopard tourism within the region has been growing haphazardly without any prior visioning and we hope this habitat suitability model can be used as an aid for villages and tour operators for the future growth and more sustainable distribution of snow leopard tourism within Ladakh.

### Conclusion

This model included six environmental variables and showed that approximately one third of Ladakh's *c*. 90,000 km^2^ geographical area is either of high or medium suitability for snow leopards. We identified areas with elevation ranging from 2,800 m to 4,600 m and ruggedness from 450 m to 1,800 m as highly suitable snow leopard habitat, given that all other environmental variables are also within favourable ranges including the high habitat suitability of prey. We propose that the snow leopard habitat suitability model be used by conservationists to better identify optimal locations for human-wildlife conflict mitigation efforts such as livestock corrals and winter homestays to increase the value of snow leopards and reduce occurrence of retaliatory killing.

## Supporting information

S1 Fig Combined prey (Asiatic ibex and blue sheep) suitability map of Ladakh showing presence locations (n = 90) used in MaxEnt model construction. (TIF)

S2 Fig Measure of training gain, test gain, or AUC value when the combined prey MaxEnt model was constructed with a single environmental variable. (TIF)

S3 Fig Marginal response curves of environmental variables on prey habitat suitability (mean ± one standard deviation) reproduced from MaxEnt across the realised range of values within Ladakh. Landcover classes; 11 (Post-flooding or irrigated croplands), 14 (Rainfed croplands), 20 (Mosaic cropland), 30 (Mosaic vegetation), 110 (Mosaic forest or shrubland), 120 (Mosaic grassland/forest or shrubland), 140 (Closed to open herbaceous vegetation), 150 (Sparse vegetation), 200 (Bare areas), 210 (Water bodies), 220 (Permanent snow and ice).
(TIF)

S4 Fig Individual response curves of environmental variables on snow leopard habitat suitability (mean ± one standard deviation) reproduced from MaxEnt across the realised range of values within Ladakh. Landcover classes; 11 (Post-flooding or irrigated croplands), 14 (Rainfed croplands), 20 (Mosaic cropland), 30 (Mosaic vegetation), 110 (Mosaic forest or shrubland), 120 (Mosaic grassland/forest or shrubland), 140 (Closed to open herbaceous vegetation), 150 (Sparse vegetation), 200 (Bare areas), 210 (Water bodies), 220 (Permanent snow and ice).
(TIF)

S5 Fig Measure of training gain, test gain, or AUC value when the snow leopard MaxEnt model was constructed with a single environmental variable. (TIF)

S6 Fig Marginal response curves of environmental variables on snow leopard habitat suitability (mean ± one standard deviation) reproduced from MaxEnt across the realised range of values within Ladakh. Landcover classes; 11 (Post-flooding or irrigated croplands), 14 (Rainfed croplands), 20 (Mosaic cropland), 30 (Mosaic vegetation), 110 (Mosaic forest or shrubland), 120 (Mosaic grassland/forest or shrubland), 140 (Closed to open herbaceous vegetation), 150 (Sparse vegetation), 200 (Bare areas), 210 (Water bodies), 220 (Permanent snow and ice).
(TIF)

S7 Fig Individual response curves of environmental variables on snow leopard habitat suitability (mean ± one standard deviation) reproduced from MaxEnt across the realised range of values within Ladakh. Landcover classes; 11 (Post-flooding or irrigated croplands), 14 (Rainfed croplands), 20 (Mosaic cropland), 30 (Mosaic vegetation), 110 (Mosaic forest or shrubland), 120 (Mosaic grassland/forest or shrubland), 140 (Closed to open herbaceous vegetation), 150 (Sparse vegetation), 200 (Bare areas), 210 (Water bodies), 220 (Permanent snow and ice).
(TIF)

S1 Table Contribution and permutation importance values of each environmental variable in the prey model. (TIF)

S1 File Snow leopard presence locations (n = 83) included in model construction, including GPS location and method of data collection (46 from camera traps and 37 direct observations). (PDF)

S2 File Prey presence locations included in model construction (n = 84), including GPS location and method of data collection (11 from camera traps and 73 direct observations). (PDF)

S3 File Locations of villages (n = 325), depredation events (n = 80), predator-proof corrals (n = 130), and homestays (n = 125) with corresponding snow leopard habitat suitability. (PDF)
