# Supplementary figures and images for: Modelling potential habitat for snow leopards (Panthera uncia) in Ladakh, India
Source: PLoS One. 2019 Jan 29;14(1):e0211509. doi: 10.1371/journal.pone.0211509 (PMC6350993; doi:10.1371/journal.pone.0211509)

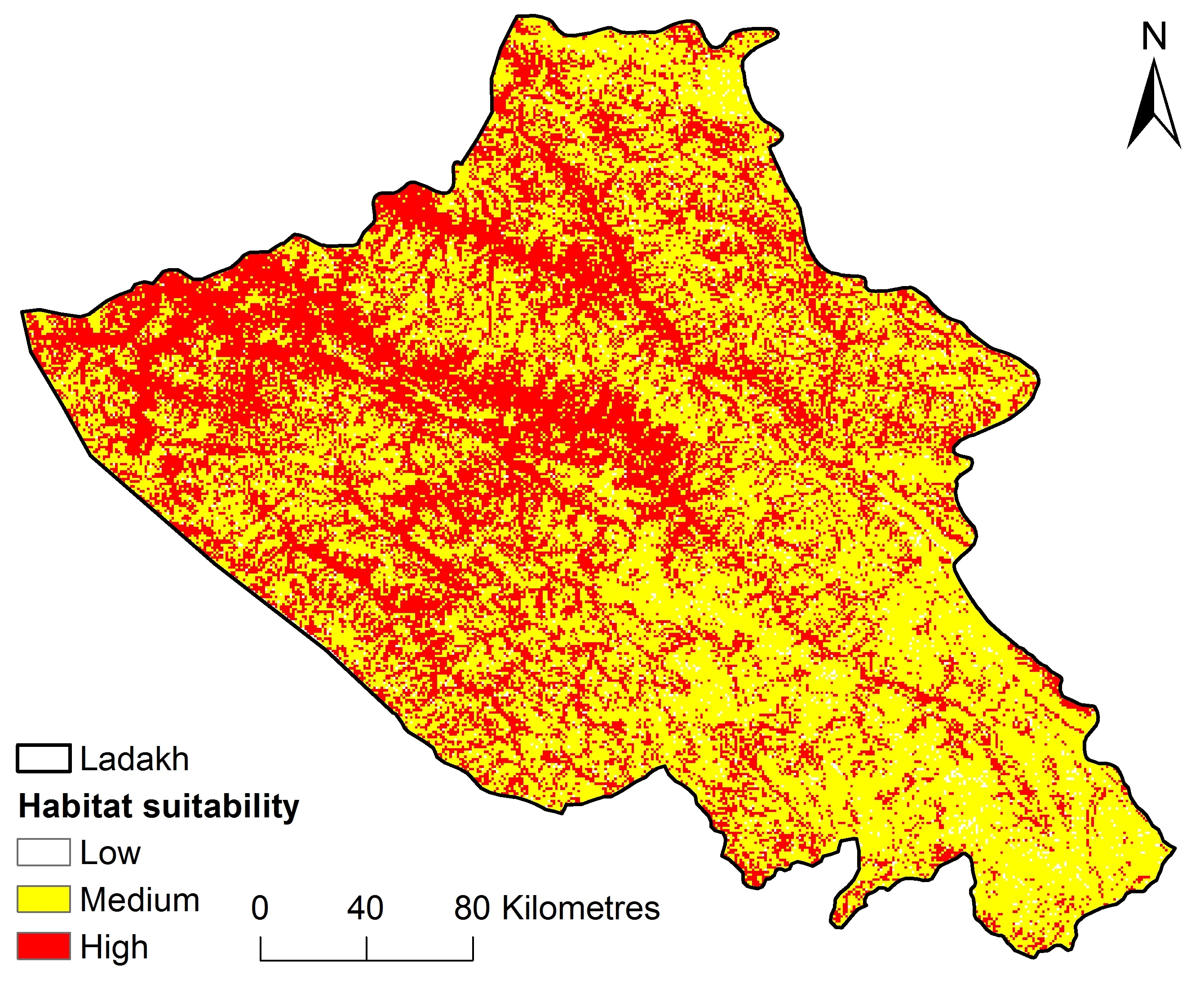

Supplement: S1 Fig — (TIF) [file pone.0211509.s001.tif]

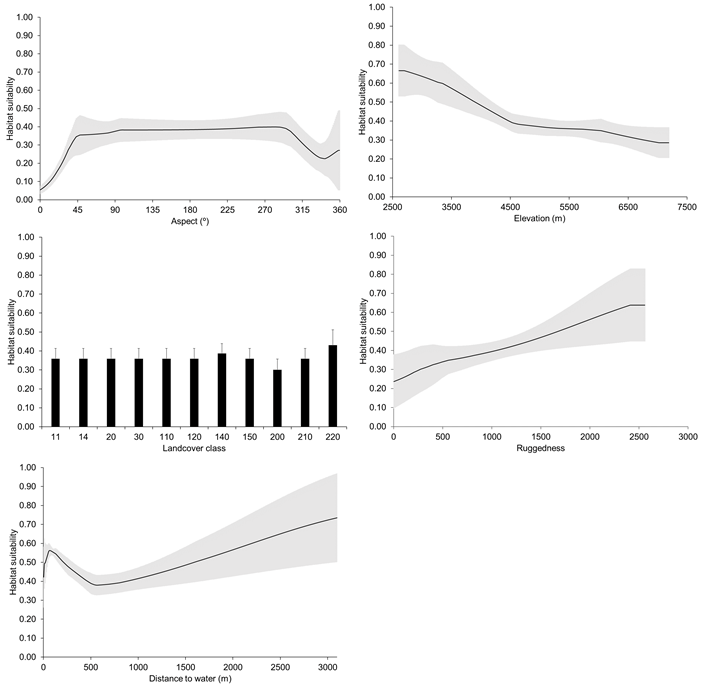

Supplement: S3 Fig — Landcover classes; 11 (Post-flooding or irrigated croplands), 14 (Rainfed croplands), 20 (Mosaic cropland), 30 (Mosaic vegetation), 110 (Mosaic forest or shrubland), 120 (Mosaic grassland/forest or shrubland), 140 (Closed to open herbaceous vegetation), 150 (Sparse vegetation), 200 (Bare areas), 210 (Water bodies), 220 (Permanent snow and ice). (TIF) [file pone.0211509.s003.tif]

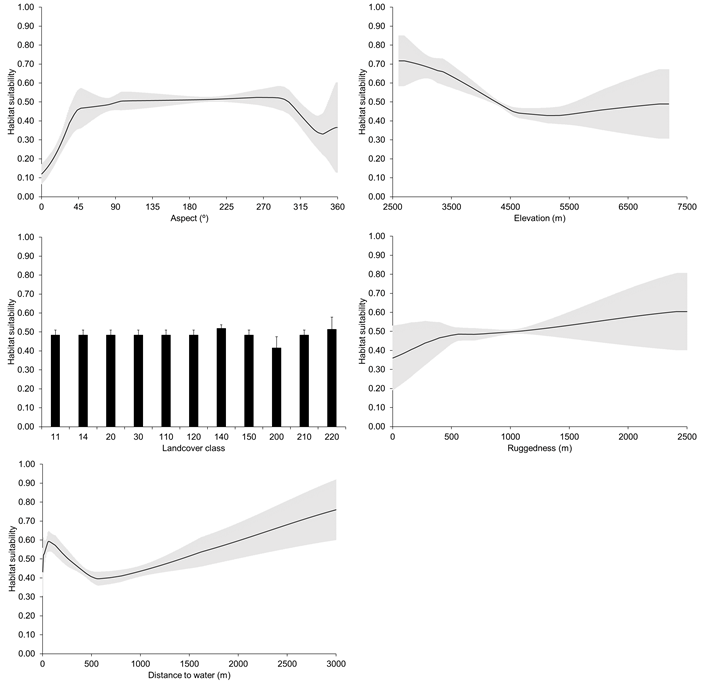

Supplement: S4 Fig — Landcover classes; 11 (Post-flooding or irrigated croplands), 14 (Rainfed croplands), 20 (Mosaic cropland), 30 (Mosaic vegetation), 110 (Mosaic forest or shrubland), 120 (Mosaic grassland/forest or shrubland), 140 (Closed to open herbaceous vegetation), 150 (Sparse vegetation), 200 (Bare areas), 210 (Water bodies), 220 (Permanent snow and ice). (TIF) [file pone.0211509.s004.tif]

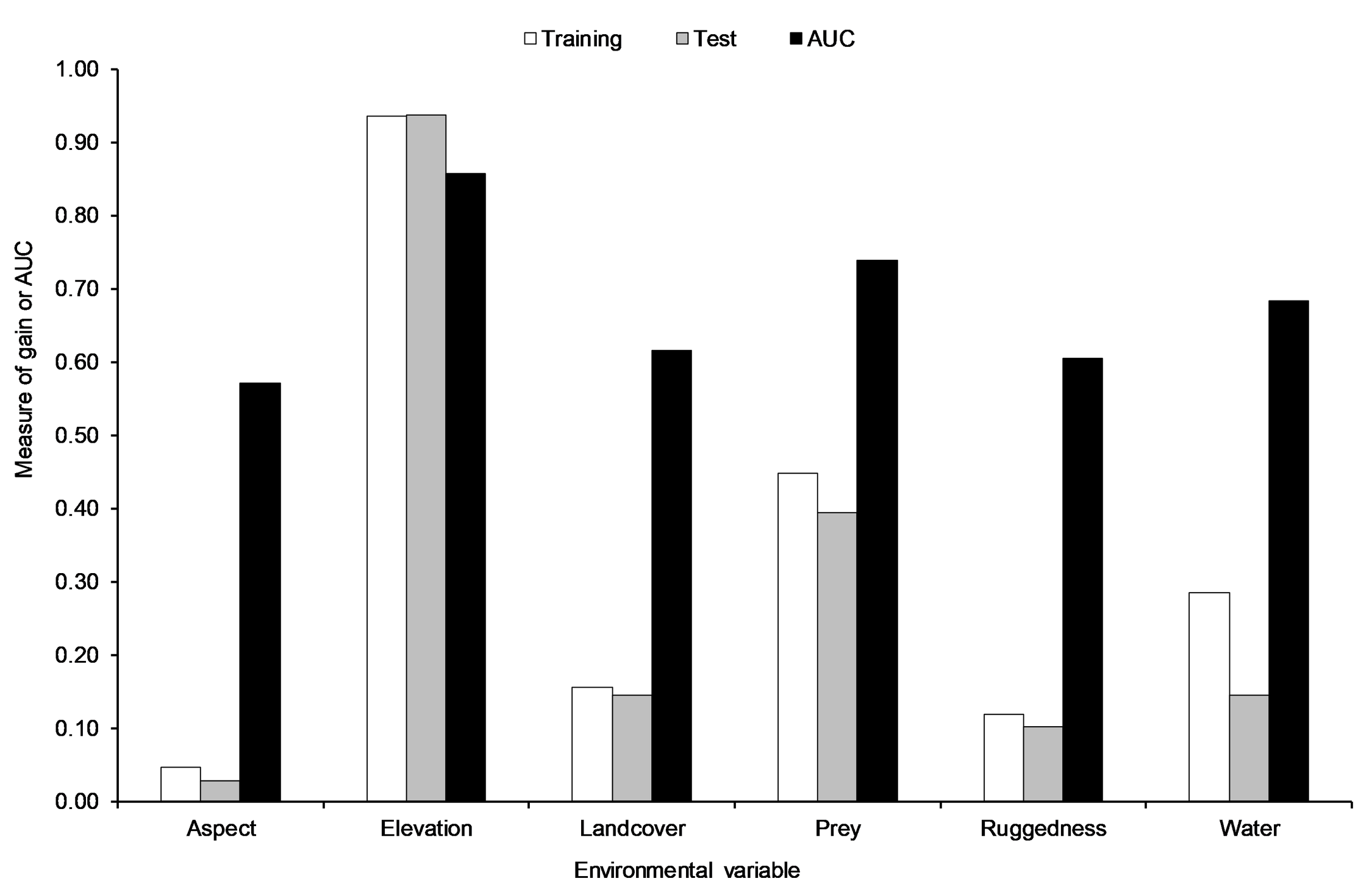

Supplement: S5 Fig — (TIF) [file pone.0211509.s005.tif]

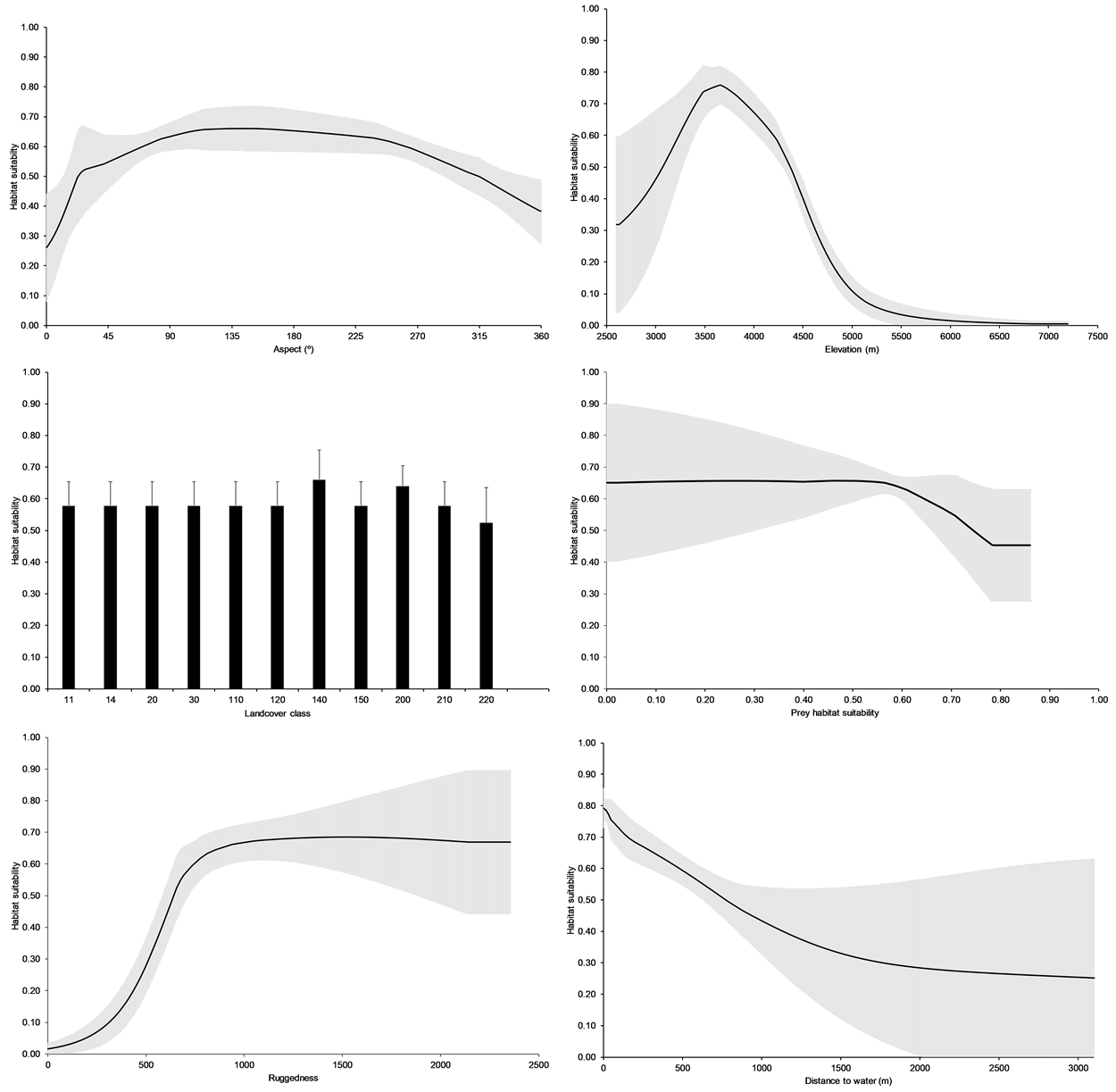

Supplement: S6 Fig — Landcover classes; 11 (Post-flooding or irrigated croplands), 14 (Rainfed croplands), 20 (Mosaic cropland), 30 (Mosaic vegetation), 110 (Mosaic forest or shrubland), 120 (Mosaic grassland/forest or shrubland), 140 (Closed to open herbaceous vegetation), 150 (Sparse vegetation), 200 (Bare areas), 210 (Water bodies), 220 (Permanent snow and ice). (TIF) [file pone.0211509.s006.tif]

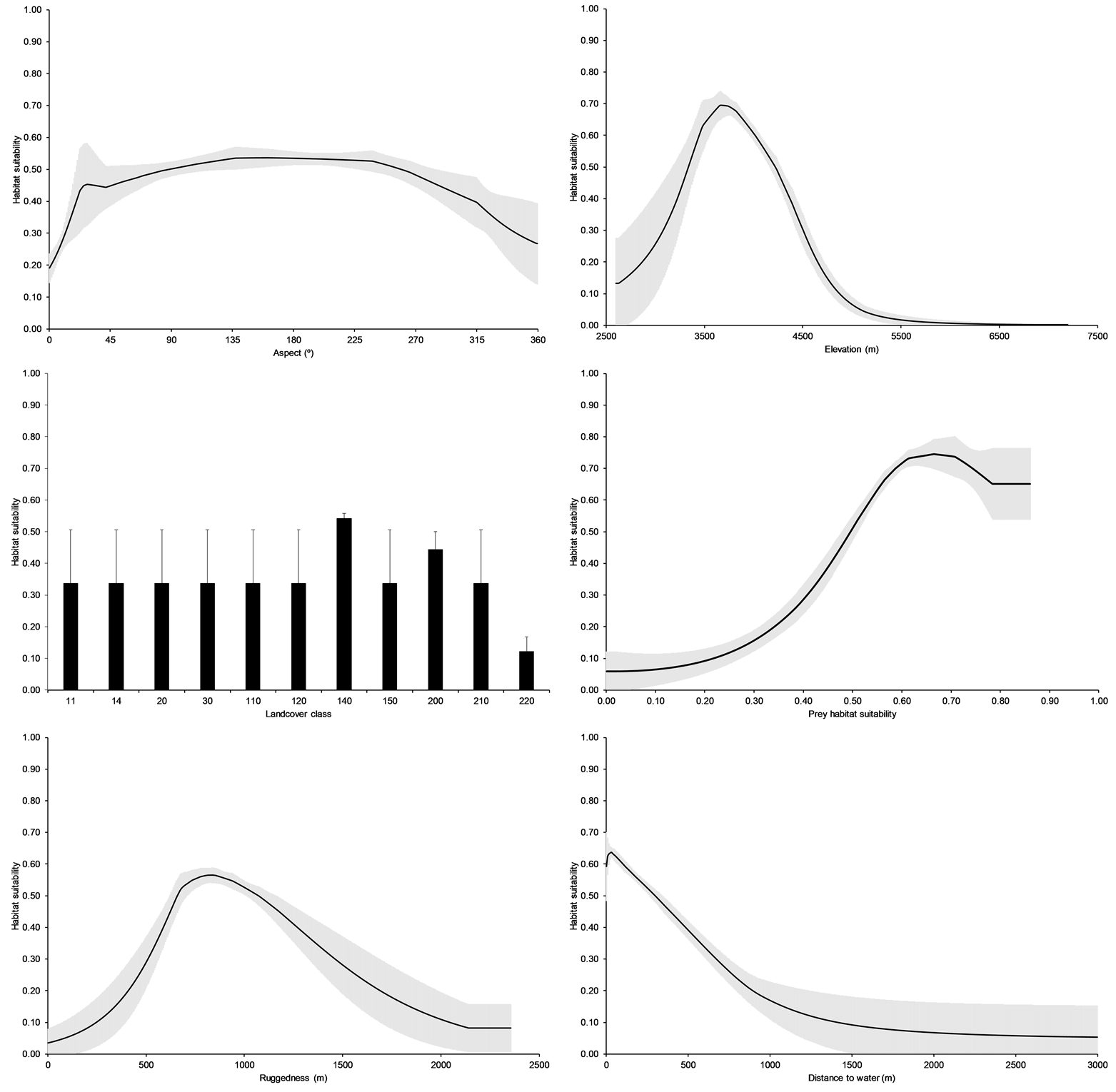

Supplement: S7 Fig — Landcover classes; 11 (Post-flooding or irrigated croplands), 14 (Rainfed croplands), 20 (Mosaic cropland), 30 (Mosaic vegetation), 110 (Mosaic forest or shrubland), 120 (Mosaic grassland/forest or shrubland), 140 (Closed to open herbaceous vegetation), 150 (Sparse vegetation), 200 (Bare areas), 210 (Water bodies), 220 (Permanent snow and ice). (TIF) [file pone.0211509.s007.tif]

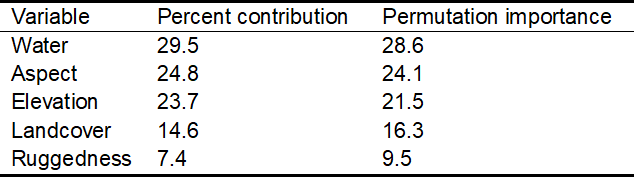

Supplement: S1 Table — (TIF) [file pone.0211509.s008.tif]
