# Supplementary material for: Modelling potential habitat for snow leopards (Panthera uncia) in Ladakh, India
Source: PLoS One. 2019 Jan 29;14(1):e0211509. doi: 10.1371/journal.pone.0211509 (PMC6350993; doi:10.1371/journal.pone.0211509)
Supplement: S1 File — (PDF) [file pone.0211509.s009.pdf]

	Deg WGS84		
Species	Latitude	Longitude	Sample
Panthera uncia	33.5531	78.0845	Camera trap
Panthera uncia	33.5692	78.1653	Camera trap
Panthera uncia	33.5743	77.0249	Camera trap
Panthera uncia	33.5791	78.1245	Camera trap
Panthera uncia	33.5856	78.1396	Camera trap
Panthera uncia	33.6229	77.9844	Camera trap
Panthera uncia	33.6339	78.0148	Camera trap
Panthera uncia	33.6412	78.0010	Camera trap
Panthera uncia	33.6485	77.0131	Camera trap
Panthera uncia	33.6491	77.0371	Camera trap
Panthera uncia	33.6560	77.0703	Camera trap
Panthera uncia	33.6657	77.9622	Camera trap
Panthera uncia	33.6833	76.9683	Camera trap
Panthera uncia	33.6873	77.9267	Camera trap
Panthera uncia	33.6996	76.9436	Camera trap
Panthera uncia	33.7036	76.9177	Camera trap
Panthera uncia	33.7136	77.9422	Camera trap
Panthera uncia	33.7265	76.8654	Camera trap
Panthera uncia	33.7422	77.9020	Camera trap
Panthera uncia	33.7513	77.9252	Camera trap
Panthera uncia	33.7630	77.9878	Camera trap
Panthera uncia	33.7653	77.9396	Camera trap
Panthera uncia	33.7660	78.0204	Camera trap
Panthera uncia	34.2731	77.1617	Camera trap
Panthera uncia	34.2907	77.1861	Camera trap
Panthera uncia	34.3038	77.3033	Camera trap
Panthera uncia	34.3097	76.9833	Camera trap
Panthera uncia	34.3264	77.1605	Camera trap
Panthera uncia	34.3299	76.9374	Camera trap
Panthera uncia	34.3303	77.0397	Camera trap
Panthera uncia	34.3322	77.0924	Camera trap
Panthera uncia	34.3324	77.2423	Camera trap
Panthera uncia	34.3336	77.1816	Camera trap
Panthera uncia	34.3421	77.0352	Camera trap
Panthera uncia	34.3443	77.1964	Camera trap
Panthera uncia	34.3468	77.1307	Camera trap
Panthera uncia	34.3611	76.7421	Camera trap
Panthera uncia	34.3658	76.9482	Camera trap
Panthera uncia	34.3701	76.7921	Camera trap
Panthera uncia	34.3709	77.2962	Camera trap
Panthera uncia	34.3719	77.7519	Camera trap
Panthera uncia	34.3746	76.7382	Camera trap

Panthera uncia	34.3778	77.0015	Camera trap
Panthera uncia	34.3789	77.0520	Camera trap
Panthera uncia	34.3837	76.9517	Camera trap
Panthera uncia	34.3906	76.7485	Camera trap
Panthera uncia	33.5810	78.0915	Observation
Panthera uncia	33.6528	76.9910	Observation
Panthera uncia	33.8007	77.6214	Observation
Panthera uncia	34.0210	77.5098	Observation
Panthera uncia	34.0258	77.5235	Observation
Panthera uncia	34.0277	77.5220	Observation
Panthera uncia	34.0289	77.5023	Observation
Panthera uncia	34.0302	77.5066	Observation
Panthera uncia	34.0362	77.5183	Observation
Panthera uncia	34.0371	77.5389	Observation
Panthera uncia	34.0400	77.4132	Observation
Panthera uncia	34.0447	77.4990	Observation
Panthera uncia	34.0455	77.5317	Observation
Panthera uncia	34.0499	77.4056	Observation
Panthera uncia	34.0562	77.4192	Observation
Panthera uncia	34.0613	77.4510	Observation
Panthera uncia	34.0646	77.4366	Observation
Panthera uncia	34.0652	77.4164	Observation
Panthera uncia	34.0761	77.4412	Observation
Panthera uncia	34.0767	77.4270	Observation
Panthera uncia	34.0827	77.4117	Observation
Panthera uncia	34.0829	77.3921	Observation
Panthera uncia	34.0844	77.4235	Observation
Panthera uncia	34.0865	77.4059	Observation
Panthera uncia	34.0989	77.4186	Observation
Panthera uncia	34.1255	77.4294	Observation
Panthera uncia	34.3173	77.1188	Observation
Panthera uncia	34.3198	77.1676	Observation
Panthera uncia	34.3258	77.1326	Observation
Panthera uncia	34.3259	77.1479	Observation
Panthera uncia	34.3269	77.1218	Observation
Panthera uncia	34.3279	76.9957	Observation
Panthera uncia	34.3350	77.1285	Observation
Panthera uncia	34.3417	77.1259	Observation
Panthera uncia	34.3430	77.1131	Observation
Panthera uncia	34.3501	77.1453	Observation
Panthera uncia	34.7367	77.9889	Observation
