# Supplementary material for: Modelling potential habitat for snow leopards (Panthera uncia) in Ladakh, India
Source: PLoS One. 2019 Jan 29;14(1):e0211509. doi: 10.1371/journal.pone.0211509 (PMC6350993; doi:10.1371/journal.pone.0211509)
Supplement: S2 File — (PDF) [file pone.0211509.s010.pdf]

	Deg WGS84		
Species	Latitude	Longitude	Sample
Capra sibirica	33.7015	76.9405	Camera trap
Capra sibirica	33.7265	76.8654	Camera trap
Capra sibirica	34.2695	77.1859	Camera trap
Capra sibirica	34.2861	77.1693	Camera trap
Capra sibirica	34.3030	77.1497	Camera trap
Capra sibirica	34.3097	76.9833	Camera trap
Capra sibirica	34.3314	76.8841	Camera trap
Capra sibirica	34.3339	77.1022	Camera trap
Capra sibirica	34.3443	77.1964	Camera trap
Capra sibirica	34.3709	77.2962	Camera trap
Capra sibirica	34.3729	77.2011	Camera trap
Capra sibirica	33.0058	77.1728	Observation
Capra sibirica	33.1143	77.1749	Observation
Capra sibirica	33.1656	77.0703	Observation
Capra sibirica	33.1656	77.1749	Observation
Capra sibirica	33.1656	77.2322	Observation
Capra sibirica	33.4339	77.2322	Observation
Capra sibirica	33.5937	76.3446	Observation
Capra sibirica	34.1023	76.4553	Observation
Capra sibirica	34.1023	77.2322	Observation
Capra sibirica	34.1138	76.1880	Observation
Capra sibirica	34.1515	76.4040	Observation
Capra sibirica	34.1617	77.0724	Observation
Capra sibirica	34.1649	76.8130	Observation
Capra sibirica	34.2159	76.2946	Observation
Capra sibirica	34.2644	76.3941	Observation
Capra sibirica	34.2724	76.4553	Observation
Capra sibirica	34.3154	78.1649	Observation
Capra sibirica	34.3246	76.4033	Observation
Capra sibirica	34.3639	76.8130	Observation
Capra sibirica	34.3750	76.1257	Observation
Capra sibirica	34.3809	78.1157	Observation
Capra sibirica	34.3890	76.5617	Observation
Capra sibirica	34.4301	78.0051	Observation
Capra sibirica	34.4752	78.1034	Observation
Capra sibirica	34.4855	77.3347	Observation
Capra sibirica	34.4916	77.2813	Observation
Capra sibirica	34.5346	76.5617	Observation
Capra sibirica	34.5346	77.0724	Observation
Capra sibirica	34.5360	76.1257	Observation
Capra sibirica	34.5817	76.5126	Observation
Capra sibirica	35.0001	77.2014	Observation

Capra sibirica	35.0001	77.1809	Observation
Capra sibirica	35.0453	77.3347	Observation
Capra sibirica	35.1436	77.3407	Observation
Capra sibirica	35.1538	77.2916	Observation
Capra sibirica	35.2051	77.2322	Observation
Capra sibirica	35.3034	77.1809	Observation
Pseudois nayaur	34.2282	77.0149	Observation
Pseudois nayaur	33.0055	78.4929	Observation
Pseudois nayaur	33.0055	78.3842	Observation
Pseudois nayaur	33.0058	77.5005	Observation
Pseudois nayaur	33.0540	78.4327	Observation
Pseudois nayaur	33.0549	78.1649	Observation
Pseudois nayaur	33.0549	77.5005	Observation
Pseudois nayaur	33.1204	77.3859	Observation
Pseudois nayaur	33.1656	78.1649	Observation
Pseudois nayaur	33.1718	78.3751	Observation
Pseudois nayaur	33.2137	78.5531	Observation
Pseudois nayaur	33.2203	78.3751	Observation
Pseudois nayaur	33.2250	77.5559	Observation
Pseudois nayaur	33.2713	78.2821	Observation
Pseudois nayaur	33.2741	77.3347	Observation
Pseudois nayaur	33.3848	77.3347	Observation
Pseudois nayaur	33.3848	77.4453	Observation
Pseudois nayaur	33.3848	77.5457	Observation
Pseudois nayaur	33.3852	78.2821	Observation
Pseudois nayaur	33.4339	78.3718	Observation
Pseudois nayaur	33.4428	78.2704	Observation
Pseudois nayaur	33.5449	78.1709	Observation
Pseudois nayaur	33.5934	78.2245	Observation
Pseudois nayaur	34.0040	77.5005	Observation
Pseudois nayaur	34.0532	77.3407	Observation
Pseudois nayaur	34.1256	78.2704	Observation
Pseudois nayaur	34.1557	78.0556	Observation
Pseudois nayaur	34.1617	77.4351	Observation
Pseudois nayaur	34.1617	77.5005	Observation
Pseudois nayaur	34.1638	78.3329	Observation
Pseudois nayaur	34.2724	77.3407	Observation
Pseudois nayaur	34.2724	78.4435	Observation
Pseudois nayaur	35.0001	78.0051	Observation
Pseudois nayaur	35.1457	77.5005	Observation
Pseudois nayaur	35.2051	77.4453	Observation
Pseudois nayaur	35.2051	77.5457	Observation
