# Supplementary material for: Modelling potential habitat for snow leopards (Panthera uncia) in Ladakh, India
Source: PLoS One. 2019 Jan 29;14(1):e0211509. doi: 10.1371/journal.pone.0211509 (PMC6350993; doi:10.1371/journal.pone.0211509)
Supplement: S3 File — (PDF) [file pone.0211509.s011.pdf]

	Deg WGS84					
Village	Latitude	Longitude	Corral	Depredation	Homestay	Habitat Suitability
Abran	33.6974	76.5647				1
Achambur	34.0901	75.9376				3
Akchamal	34.5557	76.1624				2
Akshow	33.7167	76.5170				1
Alchi	34.2231	77.1730				2
Amdi-Tangyar	34.2466	77.8584				3
Ang	34.3328	77.0218		2	8	2
Ang-Temisgam	34.3338	77.0215	1			2
Anmu	33.2473	77.0738			2	3
Apati	34.5695	76.2117				2
Ating	33.5235	76.7525				1
Barchey	34.5745	76.2320				2
Barsoo	34.5445	76.1363				1
Barto	34.1807	76.1532				2
Basgo	34.2122	77.2826				3
Basgo-tunglung	34.2387	77.2822		1		3
Batambis	34.3729	76.1835				2
Biambiat	34.4320	75.8178				1
Bodhkharbu	34.2553	76.6351				2
Bogdang	34.8035	77.0421				3
Chah	33.2408	77.1595	19		5	3
Chamshen Charasha	34.6774	77.5514				1
Changa	33.9360	77.7241				2
Changlung	34.9301	77.4726				2
Chemrey	33.9500	77.7807				2
Chiktan	34.4669	76.5120				2
Chiling Sumda	34.0388	77.2031			4	2
Choglamsar	34.0995	77.5965				1
Chokial	34.4216	75.9061				3
Choskar	34.4035	76.1423				1
Chosker Suru	34.0983	76.0079				3
Chuchot Gongma	34.0367	77.6518				1
Chuchot Shama	34.0607	77.6225				1
Chuchot Yokma	34.0805	77.5996				1
Chulichan	34.4299	76.5221				3
Chuliskambo	34.5308	76.1010				3
Chulungkha	34.8175	76.9563				1
Chumathang	33.3600	78.3405				1
Chumur	32.6667	78.5928				2
Chushul	33.6021	78.6526				1
Dah	34.6047	76.5089				3
Darkiat	34.4458	76.2801				3
Debring	33.4103	77.8863				1
Derchiks	34.5505	76.4365				3
Dethang	33.1879	77.1615	1			3
Diger	34.2941	77.8062	1			3
Disket	34.5496	77.5517				1
Domkhar	34.3916	76.7738				2

Dorzong	33.2916	77.0168	2			3
Dungti	33.1923	78.9581				1
Durbuk	34.0961	78.1222				3
Echer	33.3056	76.9974	3		4	3
Farona	34.3416	75.9758				3
Garashisa	33.2763	77.0316	1			3
Garkon	34.5647	76.4817				3
Gera-Mangu	34.2457	77.1640				1
Gia	33.6493	77.7393				3
Gindial	34.4142	75.8570				3
Gogra	34.2987	78.9459				2
Goshan	34.4061	75.7990				3
Gundmangalpur	34.3528	76.0610				1
Gyaling	34.1872	75.9482				3
Hagnis	34.5139	76.4946				3
Hamiling	33.6684	76.6254				1
Hankar-Doltokling-Umlung	33.7793	77.5137	5			3
Hanley	32.7900	78.9997				2
Hanu	34.5901	76.6221				2
Hanupata	34.1608	76.8415	1			3
Hardas	34.6002	76.1055				2
Haripora	34.4194	75.8279				3
Hemis	33.9118	77.7059				3
Hemisshukpachan	34.3162	77.0773	1	6	4	2
Hemya	33.6543	78.0083			4	3
Heniskot	34.3074	76.6203				2
Hepti	34.2613	77.0089				3
Holiyal	34.4378	75.7630				1
Hunder	34.5890	77.4717				1
Hunder Dok	34.4929	77.4325				3
Hundri	34.6705	77.3883				1
Icher	33.1934	77.1802				2
Igoo	33.9037	77.8105				2
Itchoo	34.1326	76.2903				1
Jusgund	34.3827	75.9647				3
Kaksar	34.5193	75.9732				3
Kanji	34.2324	76.6055				3
Kanor	34.3692	76.1093				3
Karamba	34.3842	76.2501				2
KarcheY Khar	34.2259	76.0028				3
Kargaim	33.8791	78.2842				2
Kargi	34.1317	75.9395				3
Kargyak	33.0625	77.2247				2
Karit	34.4658	76.3898				3
Karkit	34.5426	76.0514				2
Karpo Khar	34.2531	75.9681				2
Karsha	33.5317	76.9047			6	3
Karshah	33.4961	77.0017				2
Kaya	33.9877	77.2511			2	3
Keray	33.4776	78.1259				3

Kesar	33.4059	78.2384			4	1
Khachey	34.3397	76.3136				3
Khalsar	34.4909	77.7039				2
Khaltsi	34.3221	76.8812				3
Khandi	34.2385	76.0718				2
Kharbu	34.5425	75.9899				2
Khardong	34.4012	77.6575				2
Kharnak	33.2575	77.7030				1
Kharoo	33.9246	77.7351				3
Khatpoo	33.6717	77.9763	12			3
Khema	34.2456	77.8081				1
Khema Khungru	34.2838	77.8284				3
Khemi	34.9442	77.4249				2
Khows	34.1507	75.9712				3
Khuldo	32.7703	78.9844				1
Kochik	34.0387	75.9536				3
Korzok	32.9661	78.2630				2
Kubet	34.8183	77.4964				2
Kuksho	34.3783	76.5866				2
Kukste	34.4543	76.3462				2
Kumdok	33.5207	78.1886				2
Kumic	33.4969	76.9533	1			2
Kungiam	33.1994	78.6448				1
Kuru Lungnak	33.1613	77.1849	1			3
Kuyul	32.8853	79.1997				2
Kyalbok	33.2371	77.1097	1			3
Lakjung	34.5856	77.6195				1
Lalung	34.5845	76.2957				2
Lamayuru	34.2835	76.7729	1			3
Lamsusando	34.4903	76.4223				3
Langkor	33.9187	77.8533				3
Lankarchey	34.3208	75.9588				1
Largyab	34.6190	77.1240				3
Leh Dho	34.4453	76.6837				2
Likir	34.2934	77.2150		5		3
Liktse	33.7271	77.9547			6	3
Lingshet	33.9048	76.8221				3
Lochum	34.4109	76.3255				3
Lukung	33.9992	78.4077				2
Lungmi Rezing	33.4774	76.9731				2
Lyoma	33.1702	78.8234				1
Mahe	33.2615	78.4983				3
Malbekh	34.3231	76.3981				2
Mangue	34.2323	77.0487				3
Man-Pangong	33.8559	78.5275				1
Markha	33.8868	77.4234	1			3
Markha-Hankar-Umlung	33.7879	77.5952				1
Martselang	33.9065	77.7379				1
Matayan	34.3585	75.6256				3
Matho	33.9958	77.6346	16			1

Minji	34.4450	76.0946			3
Miru	33.7404	77.7575			3
Mudh	33.2021	78.6956			1
Murad Bagh	34.4258	75.8030			3
Murgo	35.0410	77.9375			2
Mushku	34.4378	75.6659			2
Nagmakusar	34.2881	76.0134			2
Namsuru	34.1142	76.0226			1
Nang	34.0452	77.7421			3
Ney	34.2788	77.3001			2
Nimoo	34.1932	77.3419			2
Nunamchey	34.4319	76.2881			3
Nurla	34.3024	76.9833			3
Nyoma	33.2042	78.6488			1
Nyrags	33.8765	76.9269	6		3
Padam	33.4041	76.9072			3
Panamik	34.7961	77.5263			1
Pandras	34.4115	75.6332			3
Pang	33.1292	77.7826			2
Pangkumik	33.5012	76.9586	1		2
Panikhar	34.1133	75.9492			3
Parkachik	34.0528	76.0017			1
Partapur	34.6100	77.4548			1
Phey	34.1317	77.4678	1		2
Phobrang	34.0540	78.4420			1
Phoo	34.3821	76.2313			3
Photoksar	34.0709	76.8294	4		3
Phuktal	33.2689	77.1807		1	3
Phuktse	33.8209	77.9099			3
Phultuks	34.5604	76.0921			3
Phyang	34.1863	77.4895			2
Pibiting	33.4803	76.8912			1
Pidmo	33.6903	76.9328	1	5	2
Pipcha	33.3455	76.9920	1		1
Pishu	33.6328	76.9836	1	5	2
Poyan	34.5624	76.1327			1
Pranti	34.1170	75.9405			3
Puga	33.2284	78.2992			1
Punguk	32.7581	78.8935			1
Purtikchy	34.1706	75.9251			3
Push Kum	34.5116	76.1929			2
Ralakung	33.7511	76.6955	1		2
Rambirpur (Drass)	34.4141	75.7395			3
Ranbirpur	34.0284	77.6798			1
Rangdum	34.0369	76.3741			1
Rantaq Shah	33.5628	76.7539			2
Raru	33.3051	76.9992		6	3
Remala Skyagam	33.6359	76.6734			2
Rhongo	33.1150	78.8356			1
Rinam	33.5411	76.9590	1		2

Rumbak	34.0545	77.4318	1		9	3
Ruru Moony	33.2861	76.9854				2
Saboo	34.1284	77.6272	8			2
Safi	34.2966	76.2493				2
Sakti	33.9928	77.8148	1			1
Salapi Ruruk	33.4920	76.8612				1
Saleskot	34.3669	76.0044				3
Samad-Rockchen	33.2977	77.9066				1
Samrah	34.4110	76.5235				1
Sangra	34.2238	75.9739				1
Sangtha	33.3173	77.7043				1
Sanjak	34.5780	76.5271				3
Saspochey	34.3119	77.1591	6	8	8	3
Saspol	34.2461	77.1639				1
Satho Kargyam	33.9072	78.2657				1
Selapigai Pak	33.4308	76.8758				3
Seni	33.4458	76.8264				1
Shachukul	33.9866	78.1066				2
Shadey	33.3778	77.2527	1			3
Shakar	34.4147	76.4428				3
Shan Shaday	33.4054	77.2398				2
Shang	33.8542	77.7076				3
Shara	33.7930	77.8976				2
Shargol	34.3431	76.3709				2
Sharnos	33.7730	77.8852				2
Shela Phu	33.4349	76.9348		7		3
Shergandi	34.1724	76.1823				2
Shey	34.0695	77.6335				1
Shilikchey	34.5742	76.1245				3
Shilingskit	33.5596	77.0074	1			2
Shilla	33.4304	76.8963	1			2
Shimsha	34.4548	76.0314				3
Shingo	34.0284	77.3061			1	3
Shyok	34.1794	78.1413				3
Silmo	34.6291	76.3239				3
Skambo	34.3931	76.2631				3
Skampuk	34.6246	77.4342				1
Skindiang	34.3697	76.9021			5	3
Skitmang	33.3833	78.2722				2
Sku	33.9792	77.2621			1	2
Sku-Kaya-Shingo	34.0267	77.3397	2			1
Skurbuchan	34.4343	76.7103				3
Skuru	34.6714	77.2934				1
Stakmo	34.1109	77.6959				3
Stakna	34.0042	77.6808				1
Stakpa	34.3044	75.9238				3
Staktse	34.3075	76.6030				3
Stok	34.0634	77.5483				2
Stongdey	33.5253	76.9796	1			2
Sumdo	33.2345	78.3661				1

Sumoor	34.6182	77.6185				1
Tacha	34.4270	76.3614				2
Tai Suru	34.1180	75.9467			2	3
Takmachik	34.3825	76.7642				2
Takshi	34.8888	76.8026				2
Tambis	34.4183	76.0551				1
Tampo	33.6425	76.9818	2			1
Tangday Kumi	34.3584	76.2636				3
Tangol	34.0262	75.9130				1
Tangse Kongma	33.1370	77.2140		1		2
Tangtsey	34.0330	78.1670				1
Tangyar	34.2541	77.8741	1			3
Tangze Lungnak	33.1439	77.2098	1			2
Tangzey	33.1372	77.2134	1	5		2
Tar	34.2794	76.9669				3
Tarchit	33.7034	77.9485				3
Taru	34.2050	77.4315				2
Tarutse	34.2743	77.1961		1	4	2
Taya Charchar	34.3473	76.9726		1		2
Techa Khasar	33.4792	76.8693				1
Temisgam	34.3241	76.9882			6	3
Terchey	34.6615	77.3291				1
Teri	33.5638	78.0368				2
Testa	33.1154	77.2008	1			2
Thakhan Tungri	33.5234	76.7974	1			2
Thang Dumbur	34.3071	76.0242				1
Thanga Chathang	34.9259	76.7971				2
Thangra	33.2142	78.8807				3
Thangso	33.1276	77.2139		7		3
Thasgam	34.4766	75.9343				2
Thasgam Thaine	34.3316	76.1635				3
Thiksey	34.0444	77.6695				1
Thrangos	34.3860	75.8752				1
Thukje	33.3600	78.0219				1
Thuls Pursa	34.0851	75.9772				3
Tia	34.3416	76.9755	2			2
Tiggar	34.6356	77.6147				1
Timisgam	34.3238	76.9886				3
Tingdo	34.3584	76.2636				3
Tirisha	34.7317	77.5641	1			2
Tirit	34.5403	77.6447	1			1
Tongsted	34.9848	77.3943				1
Tonrian Thagan	33.5330	76.7916				3
Toumel	34.4817	76.2800				2
Trespore	34.4226	76.0454				3
Tronjen (Trankuchan)	34.3862	75.7417				2
Tsazar	33.5874	77.0080	1			1
Tsogsti	34.0993	77.3040				3
Tsogstsalu	34.2683	78.7430				1
Tukla	33.7455	77.9856		15	6	3

Tukla phu	33.7583	78.0439		7		3
Tunglung-Basgo	34.2390	77.2844			5	2
Turtuk	34.8479	76.8257				1
Tyakshi	34.8862	76.8043				2
Udmaroo	34.7035	77.2693				2
Ukdungle	32.6013	78.9677				1
Ulley	34.3540	77.1313	3		4	1
Umba	34.2906	75.9137				3
Umla	34.2383	77.4010				2
Upshi	33.8298	77.8150				1
Upti Pipiting	33.4829	76.8896				1
Urutse	34.0430	77.4014			1	2
Wakhade	34.3689	76.4086				1
Wanla	34.2491	76.8295				1
Warisfistan	34.8734	77.1231				3
Yal	33.2095	77.1504				3
Yalboo	34.3877	75.7901				3
Yangthang Tokpo	34.3035	77.1216	2		2	2
Yogmakharbu	34.4824	76.4996				1
Yourbaltak	34.5756	76.1943				2
Yugar-Lungnak	33.2664	77.1829				3
Yulchung	33.9265	76.9125				3
Yulgok	33.2299	77.0912	1			3
Yuljuk	34.1568	75.9200				3
Zangla	33.6568	76.9842	3	14	5	2
Zangthang	33.2390	77.1217	1			3
Zingchen	33.7921	76.8156	1			3
